# Umbilical cord sparing technique of umbilicoplasty in infants with giant omphalocele

**DOI:** 10.1136/wjps-2023-000574

**Published:** 2023-07-03

**Authors:** Peng Wang, Jinfa Tou

**Affiliations:** Department of Neonatal Surgery, Zhejiang University School of Medicine Children's Hospital, Hangzhou, Zhejiang, China

**Keywords:** neonatology, technology, surgery, plastic

Omphalocele is a congenital defect of the abdominal wall characterized by the absence of abdominal muscles, fascia, and skin.^1^ According to the size of the abdominal wall defect and bulge, it can be divided into small or giant omphalocele.^2^ Surgery to repair omphalocele includes excision of the amniotic sac, individual ligation of the umbilical vessels at the level of the peritoneum, fascial closure, and reconstruction of the skin with a circumferential subcuticular purse-string suture.^3 4^ However, this technique cannot reconstruct a well-formed umbilical cord, which flattens out or disappears. This study aims to introduce an umbilical cord-sparing method to reconstruct the umbilical cord of children with giant omphalocele, thereby improving the appearance of the umbilical cord.

In this study, 14 neonates with giant omphalocele who underwent umbilicoplasty from January 2022 to June 2022 at our institution were recruited. Patient gender, birth weight, associated anomalies, size of the defect, and complications were all collected. All the children were followed up for 6 months.

The main clinical characteristics of included patients were summarized in table 1. The maximum diameter of the fascial defect ranged from 4.1 to 7.3 cm (median, 5.7 cm). There were no wound infections, and complete healing of the umbilical scars was documented during outpatient follow-up appointments.

**Table 1:** Characteristics of the included 14 patients with giant omphalocele

Case	Gender	Gestational weeks	Weight (g)	Fascial defect (cm)	Associated anomalies	Complications
1	M	37^+5^	2780	7.0	Intestinal malrotation, Meckel’s diverticulum, ASD, PDA	No
2	M	39	2680	4.5	Intestinal malrotation, ASD	No
3	M	37^+3^	3090	5.5	Intestinal malrotation	No
4	F	38^+2^	4120	4.2	Intestinal malrotation, BWS, PDA	No
5	M	37	3590	4.1	Intestinal malrotation, BWS, ASD	No
6	F	37^+2^	3050	6.3	Intestinal malrotation, ASD, VSD	No
7	F	38	3780	5.8	Intestinal malrotation, ASD	No
8	F	39	4060	6.5	Intestinal malrotation, ASD, PDA, polydactyly	No
9	M	39^+2^	3890	7.3	Intestinal malrotation, ASD, PDA, VSD	No
10	M	36^+6^	2850	4.4	Intestinal malrotation, Meckel’s diverticulum	No
11	F	38^+2^	3750	5.3	Intestinal malrotation, ASD	No
12	F	38^+4^	3960	6.8	Intestinal malrotation, ASD, PDA	No
13	M	39^+1^	3700	7.0	Intestinal malrotation, ASD, PDA	No
14	M	37^+6^	3780	5.6	Intestinal malrotation, ASD, PDA	No

ASD, atrial septal defect; BWS, Beckwith-Wiedemann syndrome; F, female; M, male; PDA, patent ductus arteriosus; VSD, ventricular septal defect.

Omphalocele is the herniation of intra-abdominal contents through the umbilical ring. Omphalocele can be divided to small and giant omphalocele depending on the size of the defect. Infants with omphalocele require to receive umbilicoplasty after birth. The reconstruction of small omphalocele is usually satisfactory, while the reconstruction of giant omphalocele is less effective.^5^ Cosmetic outcome is a common cause of parental anxiety after reconstruction of the umbilicus in neonates with giant omphalocele. There are many techniques for the reconstruction of the umbilicus.^6 7^ The results of plastic surgery are sometimes unsatisfactory due to the flattening or disappearance of the umbilical depression after surgery. We present a novel technique for umbilicoplasty during the surgical repair of giant omphalocele.

The umbilicus is a depression in the skin that indicates where the umbilical cord attachment and all layers of the abdominal wall fuse together before birth. The normal umbilical position is 60% of the distance from the inferior border of the xiphisternum to the superior border of the pubis in the midline. With the progress of society, the emergence of changing modern clothing, especially the popularity of bikini swimsuit and navel dress, there are more and more opportunities to show the umbilicus, and the esthetic value of the umbilical cord is paid more and more attention. Its absence or irregular shape can have significant psychological effects on children, especially as they enter their teenage years. Anatomically, the umbilicus is usually located at the level of the top of the iliac crests. When the umbilical cord stump falls off, the scar at the bottom of the umbilicus is concealed. The scar may be located in or near the mamelon. The mamelon can be remnants of the solid lower part of the umbilical cord, which contains the umbilical arteries and urachus. The scar pulls inward as normally occurs as a result of retraction of the umbilical arteries and urachus. By using this inward scar retraction principle, we attempted to develop an operation based on preserving the umbilical cord elements.

All patients underwent primary repair of the defect using a consistent new surgical technique. The size of the defect was measured at the time of surgery. The operation was performed under general anesthesia. Nasogastric tubes and urinary catheters were placed. The marker is marked along the midline of the body to facilitate the skin along the midline. The bulges were excised along the edge of the skin, retaining the 1 cm × 1 cm peritoneum and capsule at the root of the umbilical cord, breaking off and ligating the umbilical vein, and retaining the urethus and the umbilical artery on the second side of the umbilical cord. Then, the intestines, gallbladder, liver organs, and other organs for combined deformity were checked. The bilateral abdominal wall muscle layers were pulled back to enlarge the abdominal cavity volume and return to all the exposed organs. Absorbable suture was used to close the peritoneal layer and the muscle layer, trim the skin and free the skin and the muscle fascia layer, and close the muscle layer fascia. The suture was taken to vigorously ligate the peritoneum, capsule, urachus, and umbilical artery along the umbilical cord root. The fascia layer was stitched with 3-0 barbed lines or 5-0 sutures (figure 1).

**Figure 1:**
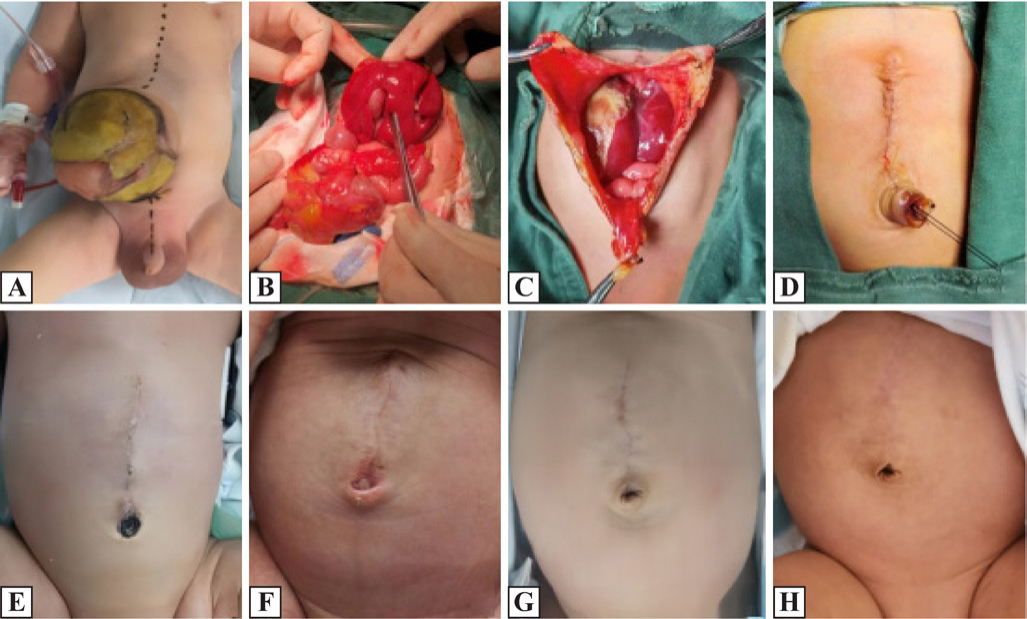
(A) Preoperative abdomen; (B) intraoperative exploration; (C) intraoperative umbilical cord preservation; (D) postoperative abdomen and postoperative umbilical morphology; (E) postoperative 2-week re-examination; (F) postoperative 4-week re-examination; (G) postoperative March re-examination; and (H) postoperative June re-examination results show that the umbilical cord is well-formed.

In this study, primary closure of the fascial defect and umbilicoplasty were performed in 14 neonates with giant omphalocele. During the procedure, the fascial defect is easily closed through the umbilical skin defect without undermining the skin from the fascial edges and umbilical cord elements. The preserved umbilical cord is ligated by a line and usually falls spontaneously 2–3 weeks after surgery. The postoperative follow-up results showed that the umbilical morphology of the child was natural-looking, with no flattening or disappearance.

One example of previous surgical methods of umbilicoplasty for treating neonatal giant omphalocele is shown in figure 2. The follow-up results indicated the flattening and disappearance of the umbilical depression.

**Figure 2:**
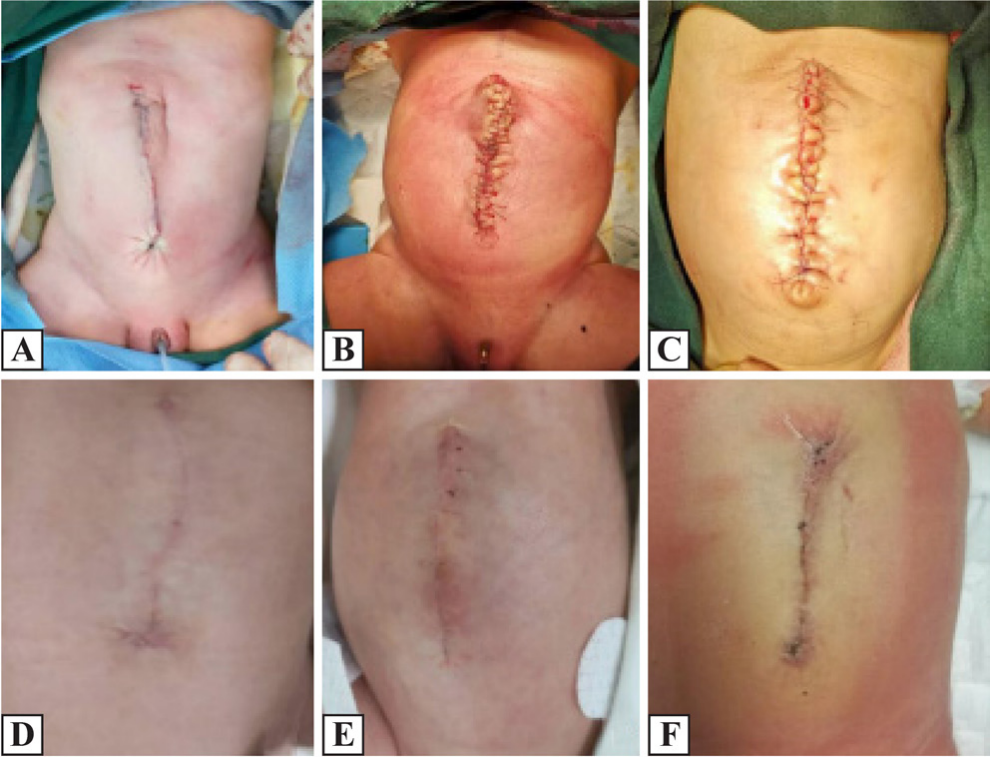
Previous surgical methods for giant omphalocele. (A–C) Postoperative abdomen and postoperative umbilical morphology. (D–F) The results suggested that the umbilical cord was not natural after surgery, and the follow-up indicated its flattening and disappearance.

In conclusion, this novel technique seems to be a simple, safe, and easily reproducible method for repairing giant omphalocele with a defect size >4 cm. This technique can retain a scarless abdomen with a very satisfying esthetic and natural-looking umbilicus. The long-term prognosis of children who received this specific technique needs to be verified by long-term follow-up in the future.

## Data Availability

All data relevant to the study are included in the article or uploaded as supplementary information.
